# Bayesian Integrative Detection of Structural Variations With False Discovery Rate Control

**DOI:** 10.1002/bimj.70128

**Published:** 2026-03-27

**Authors:** Sheng Lian, Jiandong Shi, Jingyu Hao, Zhen Zhang, Yongyi Luo, Taobo Hu, Depeng Wang, Xiaodan Fan, Shu Wang, Weichuan Yu

**Affiliations:** ^1^ Department of Electronic and Computer Engineering The Hong Kong University of Science and Technology, Clear Water Bay Hong Kong China; ^2^ Department of Statistics and Data Science The Chinese University of Hong Kong, Shatin, N.T. Hong Kong China; ^3^ Department of Breast Surgery Peking University People's Hospital Beijing China; ^4^ GrandOmics Inc. Beijing China

**Keywords:** Bayesian analysis, false discovery rate, integration, structural variation

## Abstract

Recent advances in long‐read sequencing technologies have empowered the detection of structural variations (SVs) associated with genetic diseases. Despite the availability of numerous SV callers and efforts to merge SVs from multiple tools, there remains limited research on quantifying the confidence levels for the reported results. In this work, we propose a Bayesian integration model that combines SV calls from different tools. Notably, we introduce an approach for false discovery rate (FDR) control and provide a quantitative measure for the merged SVs. Our model can handle cases where certain tools lack quality scores, showcasing its flexibility in incorporating additional tools. Through extensive simulation studies, we evaluate the performance of our method under various conditions, demonstrating the FDR estimation accuracy and improved F1 score. Furthermore, we validate our model using simulated human genome sequencing data and the HG002 dataset.

## 1 Introduction

Detecting genomic structural variations (SVs) has significantly enhanced our insights into many human diseases (Ho et al. 2020). The advent of long‐read sequencing technologies (Logsdon et al. 2020) enables us to uncover large and complex genetic variants with remarkable precision and resolution. Numerous computational methods and software tools have been instrumental in addressing the challenges associated with SV detection, including SVIM (Heller and Vingron 2019), pbsv (PacificBiosciences 2017), Sniffles (Sedlazeck et al. 2018; Smolka et al. 2024), cuteSV (Jiang et al. 2020), and DeBreak (Chen et al. 2023). These tools employ different criteria and signal extraction techniques, but no single SV caller is universally recognized as the superior choice. Therefore, integrating multiple SV detection tools to leverage their respective strengths and mitigate their limitations holds promise for improving overall performance.

Various methods are available for merging SVs from different tools. Some approaches (Dixon et al. 2018; Fang et al. 2018; Jeffares et al. 2017; Mohiyuddin et al. 2015; Wong et al. 2010) consider the intersection of SV calls from different tools as high‐confidence results. Other methods (Becker et al. 2018; Dierckxsens et al. 2021) utilize training benchmark datasets to select the best‐performing tool for specific SV types, which poses limitations when adapting to new tools. Notably, none of these methods provides a standard confidence measure for detection results, making it difficult for users to assess the reliability of the output. The R package “intansv” (Jia et al. 2020) utilizes quality scores associated with SV calls from various tools but does not primarily focus on statistical assessment. BAYSIC (Cantarel et al. 2014) employs Bayesian analysis to estimate false positive and false negative error rates when combining single‐nucleotide polymorphism (SNP) variant calls. However, in the context of SVs, it is challenging to define the negative event when there may be true variations undetected by any tool. This is because the identification of candidate positions for SVs relies on at least one tool, reporting a variation at a specific location. Consequently, there is limited research offering sufficient statistical evidence to confidently support the detected SVs and inform the process of merging results from different tools.

In this study, we propose a Bayesian integration model that combines results from different SV detection tools. To enhance the reliability and interpretability of SV calls, we utilize available quality scores and provide estimates of the false discovery rate (FDR), allowing users to output subsets of variations based on predefined thresholds. Our model also accommodates cases where certain tools lack quality scores and offers the flexibility to incorporate additional tools. Through simulation studies, we present the estimation accuracy and assess the sensitivity of our Bayesian method in multiple scenarios. Finally, we apply our model to the SV merging problem and demonstrate its advantages in two examples.

## 2 Methods

**FIGURE 1:**
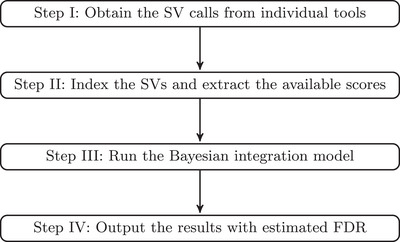
The flow chart of our method.

Figure 1 illustrates the general framework of our method. Our primary objective is to develop an integrative approach that enables FDR control. Considering that the same SV detected by different tools may exhibit slight deviations in positions and lengths, we design a tool‐aware SV merging procedure in Section 1 of the Supporting Information to effectively index the variants reported by all tools. In Section 2.1, we introduce the integration model, where we use the term “positive” to denote the detected variation for generality. We conduct Bayesian inference for parameter estimation, and the details are outlined in the Section 2 of Supporting Information. Finally, the FDR control part is provided in Section 2.2.

### 2.1 Model

Suppose we have a total of n positions obtained by merging all reported positives from J tools. Let $Y={\left\{y_{ij}\right\}}_{i=1,\cdots ,n;j=1,\cdots ,J}$ be the indicator variables, where $y_{ij}=1$ if the jth tool reports a positive at the ith position, and $y_{ij}=0$ otherwise. Additionally, we have the score variables $S={\left\{s_{ij}\right\}}_{i=1,\cdots ,n;j=1,\cdots ,J}$, representing the scores assigned by the corresponding tool at each position, with missing values possibly. We introduce latent binary variables, $Z={\left\{z_{i}\right\}}_{i=1,\cdots ,n}$, where $z_{i}=1$ indicates that the ith position is a true positive. Our goal is to infer the probability $P(z_{i}=1)$ for each position i=1,⋯,n.

Let $X={\left\{x_{i}\right\}}_{i=1,\cdots ,n}$, where $x_{i}=\sum_{j=1}^{J}y_{ij}$ denotes the number of tools reporting the ith position as positive. Since each position arises because at least one tool reports a positive, each $x_{i}$ has a minimum value of 1. Instead of assuming conditional independence among $\left(y_{i1},\cdots ,y_{iJ}\right)$, we model the joint distribution of $\left(y_{i1},\cdots ,y_{iJ},x_{i}\right)$ given $z_{i}$ as follows:

$$p\left(y_{i1},\cdots ,y_{iJ},x_{i}|z_{i}\right)=p\left(y_{i1},\cdots ,y_{iJ}|x_{i},z_{i}\right)p\left(x_{i}|z_{i}\right).$$

For the first conditional probability, we assume that the indicators, given a sum $x_{i}=k$, are independent of the underlying state $z_{i}$, that is, $p\left(y_{i1},y_{i2},\cdots ,y_{iJ}|x_{i},z_{i}\right)=p\left(y_{i1},y_{i2},\cdots ,y_{iJ}|x_{i}\right)$. This can be interpreted as a random selection without replacement from the J tools, with associated weights $v_{1k},\cdots ,v_{Jk}$, where each $v_{jk}$ represents the inclusion probability for the jth tool, and $\sum_{j=1}^{J}v_{jk}=1$. For the second term $p(x_{i}|z_{i})$, we assume two categorical distributions depending on the latent state of $z_{i}$:

$$P\left(x_{i}=k|z_{i}=1\right)=\omega_{1k},\;P\left(x_{i}=k|z_{i}=0\right)=\omega_{0k},$$

where $\sum_{k=1}^{J}\omega_{1k}=1$, $\sum_{k=1}^{J}\omega_{0k}=1$, $\omega_{11}<\omega_{12}<\cdots <\omega_{1J}$, and $\omega_{01}>\omega_{02}>\cdots >\omega_{0J}$. These monotone constraints reflect our assumption that if a position is a true positive, there is a higher probability for more tools to detect it. Conversely, this probability decreases when the position is a false positive, resembling a voting effect.

We can also make use of the score information if available. Without loss of generality, we assume a reported positive from any tool is more likely to be a true positive if the corresponding quality score is greater. However, there may be cases where some tools do not provide a score or the quality scores have varying upper and lower bounds across different tools. We denote A as the index set of the tools that provide scores, and $\left(a_{j},b_{j}\right)$ for j∈A as the predefined boundaries for the scores from the respective tool. Subsequently, we utilize Gaussian latent variables $O={\left\{o_{ij}\right\}}_{\left(i,j\right):y_{ij}=1,j\in A}$ to model the quality scores S:

$$\begin{array}{c} s_{ij}=\left\{\begin{array}{cc} a_{j}, & \;\mathrm{if}\;o_{ij}\leq a_{j}\\ o_{ij}, & \;\mathrm{if}\;a_{j}<o_{ij}<b_{j}\\ b_{j}, & \;\mathrm{if}\;o_{ij}\geq b_{j} \end{array}\right.,\\ o_{ij}|y_{ij}=1,z_{i}=1∼N\left(\mu_{1j},\sigma_{1j}^{2}\right),\\ o_{ij}|y_{ij}=1,z_{i}=0∼N\left(\mu_{0j},\sigma_{0j}^{2}\right). \end{array}$$

Here, each $s_{ij}$ follows a Gaussian distribution censored at $\left(a_{j},b_{j}\right)$, and $\mu_{1j}>\mu_{0j}$, for j∈A. The assumption that a larger score for a reported SV indicates a higher probability of being a true positive is reflected through the constraint imposed on the Gaussian mean. We only apply this score distribution for tools where the score is available, as it is equivalent to integrating out the missing scores for other tools.

**FIGURE 2:**
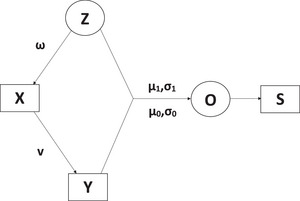
The path diagram of our model, where ovals represent the latent variables, rectangles represent the observed data, and variables indicated on the arrows represent the related parameters.

Figure 2 displays the path diagram of our Bayesian integration model.

### 2.2 Posterior Error Probability and False Discovery Rate

After running the Markov Chain Monte Carlo (MCMC) algorithm detailed in Section 2 of the Supporting Information until converged, we retrieve the posterior mean as a point estimate for $P(z_{i}=0|\cdot )$, which represents the average posterior error probability (PEP) (Käll et al. 2008) for identifying the ith position as positive. Since the expected number of false positives is equal to the sum of the PEPs, we can estimate the FDR for the jth tool as

$${\hat{\mathrm{FDR}}}_{j}=\frac{1}{\sum_{i}1_{y_{ij}=1}}\sum_{i:y_{ij}=1}P\left(z_{i}=0|\cdot \right).$$

For a cutoff threshold τ∈(0,1), we define our final decision $D_{i}$ for the ith position, where $D_{i}=1$ if $P(z_{i}=0|\cdot )<\tau$, and $D_{i}=0$ otherwise. Then, we can compute the FDR associated with the given τ as

$${\hat{\mathrm{FDR}}}_{D}\left(\tau \right)=\frac{1}{\sum_{i}1_{D_{i}=1}}\sum_{i:D_{i}=1}P\left(z_{i}=0|\cdot \right).$$

By adjusting the threshold τ, we can effectively control the FDR at various levels.

## 3 Results

### 3.1 Simulation Studies

We conducted simulation studies to assess the effectiveness of our proposed model and to check the sensitivity of its performance to various assumptions. Data were generated based on the integration model with n=2000 positions, where the true state $z_{i}$ for each position was independently determined following a binomial distribution with the probability of true positive equal to 0.7. We independently constructed five tools, each assigned an equal weight of $v_{jk}=\frac{1}{5}$ for j,k=1,…,5, suggesting that these tools do not exhibit superiority over one another.

For comparative analysis, we considered two intersection‐based methods: Vote 1 declared a positive if at least half of the tools did so, and Vote 2 required a consensus from all tools for a positive call. Our Bayesian integration model was evaluated at three FDR thresholds: 0.05, 0.01, and 0.001, corresponding to methods denoted as Model‐0.950, Model‐0.990, and Model‐0.999, respectively.

Below, we investigated three cases:

- Case 1 represented the general setting, with the voting parameters monotonically set as $\omega_{1}=\left[0.05,0.10,0.15,0.25,0.45\right]$ and $\omega_{0}=\left[0.40,0.25,0.15,0.12,0.08\right]$. We assumed that no scores are provided by the first two tools, and for j=3,4,5, we set $\mu_{1j}=2$, $\mu_{0j}=-2$, and $\sigma_{1j}=\sigma_{0j}=1$. Additionally, boundary conditions were imposed with $a_{4}=b_{5}=0$ for Tool 4 and Tool 5, respectively. Table 1 summarizes the mean and standard deviation (in parentheses) of the predicted precision from our model (1‐$\hat{{\mathrm{FDR}}_{j}}$), precision, recall, and F1 score, based on 10 replicates. We observed that the Bayesian models precisely estimated the FDR and achieved superior F1 scores in this general case.
- Case 2 addressed score mis‐specification. Building on Case 1, we introduced intentional deviations into the assumed score distributions. Specifically, when generating scores, we allowed the two components of the mixture Gaussian distribution to interchange with a probability equal to 0.10. This actually emulated the scenarios where score values might not reliably reflect the quality of positives, thus lowering the signal‐to‐noise ratio. Table 2 presents the corresponding results, suggesting that score mis‐specification can slightly impact the FDR estimates. Compared to Case 1, our Bayesian models exhibited a subtle decrease in their F1 scores but still generally outperformed the voting methods, while the two voting methods were relatively stable.
- Case 3 addressed voting mis‐specification. Building on Case 1, we mitigated the voting effect by relaxing the constraints on the parameters $\omega_{1}$ and $\omega_{0}$, which were randomly sampled from a Dirichlet(50,50,50,50,50) distribution. This could happen when multiple tools simultaneously erred due to systematic biases, such as sequencing or alignment errors, or when a certain tool exclusively identified a set of discoveries. The comparison results in Table 3 revealed that for Bayesian models, the impact of voting mis‐specification was not as substantial as the score mis‐specification, since the score information was retained. However, the performance of the intersection‐based methods was affected dramatically.

**TABLE 1:** Comparison results in the general case (Case 1). The predicted precision (1‐$\hat{{\mathrm{FDR}}_{j}}$) is very close to the empirical precision (Precision) evaluated using the truth. The bold number indicates the best F1 score.

Tool/method	1‐$\hat{{\mathrm{FDR}}_{j}}$	Precision	Recall	F1 score
Tool1	0.806 (0.012)	0.806 (0.011)	0.795 (0.007)	0.801 (0.008)
Tool2	0.804 (0.012)	0.804 (0.012)	0.791 (0.007)	0.797 (0.008)
Tool3	0.804 (0.011)	0.804 (0.012)	0.790 (0.012)	0.797 (0.011)
Tool4	0.806 (0.011)	0.806 (0.011)	0.793 (0.008)	0.799 (0.007)
Tool5	0.807 (0.012)	0.808 (0.012)	0.794 (0.013)	0.801 (0.012)
Model‐0.950	0.950 (0.000)	0.957 (0.033)	0.986 (0.011)	0.971 (0.012)
Model‐0.990	0.990 (0.000)	0.994 (0.004)	0.969 (0.008)	**0.981** (0.003)
Model‐0.999	0.999 (0.000)	0.999 (0.001)	0.936 (0.014)	0.966 (0.007)
Vote 1	0.851 (0.010)	0.851 (0.010)	0.853 (0.010)	0.852 (0.008)
Vote 2	0.926 (0.006)	0.926 (0.007)	0.455 (0.017)	0.610 (0.015)

**TABLE 2:** Comparison results in the score mis‐specification case (Case 2). The predicted precision (1‐$\hat{{\mathrm{FDR}}_{j}}$) exhibited biases in comparison to the empirical precision (Precision). The bold number indicates the best F1 score.

Tool/Method	1‐$\hat{{\mathrm{FDR}}_{j}}$	Precision	Recall	F1 score
Tool1	0.726 (0.010)	0.800 (0.009)	0.792 (0.010)	0.796 (0.007)
Tool2	0.730 (0.008)	0.802 (0.014)	0.792 (0.007)	0.797 (0.007)
Tool3	0.743 (0.007)	0.803 (0.009)	0.790 (0.010)	0.796 (0.007)
Tool4	0.724 (0.010)	0.802 (0.010)	0.794 (0.011)	0.798 (0.006)
Tool5	0.726 (0.007)	0.806 (0.010)	0.793 (0.011)	0.799 (0.009)
Model‐0.950	0.950 (0.000)	0.962 (0.006)	0.857 (0.011)	**0.907** (0.005)
Model‐0.990	0.990 (0.000)	0.976 (0.005)	0.760 (0.019)	0.854 (0.012)
Model‐0.999	0.999 (0.000)	0.979 (0.006)	0.663 (0.028)	0.790 (0.020)
Vote 1	0.773 (0.010)	0.846 (0.009)	0.850 (0.006)	0.848 (0.005)
Vote 2	0.849 (0.013)	0.926 (0.008)	0.456 (0.008)	0.611 (0.006)

**TABLE 3:** Comparison results in the voting mis‐specification case (Case 3). The bold number indicates the best F1 score.

Tool/method	1‐$\hat{{\mathrm{FDR}}_{j}}$	Precision	Recall	F1 score
Tool1	0.691 (0.020)	0.697 (0.018)	0.603 (0.013)	0.646 (0.013)
Tool2	0.697 (0.017)	0.701 (0.014)	0.601 (0.014)	0.647 (0.014)
Tool3	0.696 (0.019)	0.697 (0.019)	0.596 (0.020)	0.643 (0.018)
Tool4	0.699 (0.021)	0.701 (0.021)	0.598 (0.021)	0.646 (0.018)
Tool5	0.698 (0.021)	0.700 (0.022)	0.611 (0.018)	0.653 (0.017)
Model‐0.950	0.950 (0.000)	0.953 (0.007)	0.991 (0.004)	**0.971** (0.004)
Model‐0.990	0.990 (0.000)	0.992 (0.005)	0.899 (0.017)	0.943 (0.008)
Model‐0.999	0.999 (0.000)	0.999 (0.001)	0.792 (0.030)	0.883 (0.018)
Vote1	0.696 (0.025)	0.696 (0.025)	0.598 (0.028)	0.643 (0.024)
Vote2	0.701 (0.046)	0.701 (0.046)	0.206 (0.025)	0.318 (0.034)

Through these scenarios, we have showcased the effect of deviating from the assumptions of our model on the performance. Incorporating the scoring mechanism allows for FDR estimation, but it also has the potential to affect the accuracy of the estimates, depending on the extent of mis‐specification. Nevertheless, it is still possible to enhance the F1 score by providing information that distinguishes between the true positives and false positives.

### 3.2 Application to Merging SVs

We utilized Varsim (Mu et al. 2015) to generate gene sequences containing SVs. Specifically, we randomly selected 9418 SVs from the Database of Genomic Variants, consisting of 4972 deletions (DELs), 2,675 insertions (INSs), 1409 duplications, and 362 inversions. These SVs were inserted into the human reference genome (GRCh37). Subsequently, we simulated PacBio‐like reads by sampling long reads from the modified genome using PBSIM2 (Ono et al. 2021). The simulated sequencing reads had a mean length of 5000 base pairs (bp) with a standard deviation of 1000 bp. The accuracy of the reads was set to 95%, and the sequencing depth was set to 20×.

**FIGURE 3:**
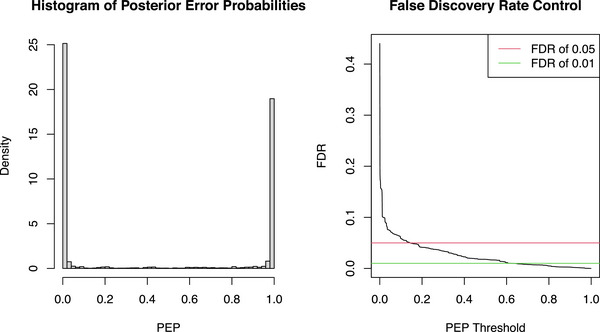
Distribution of PEPs for the total 16,044 positions and the process of FDR control.

Next, we considered five popular SV detection tools for the long‐read sequencing data: SVIM (Heller and Vingron 2019), pbsv (PacificBiosciences 2017), Sniffles (Sedlazeck et al. 2018), cuteSV (Jiang et al. 2020), and DeBreak (Chen et al. 2023). Each caller was run independently with default settings to generate corresponding variant call format (VCF) files, from which we extracted essential SV information, including the starting position, end position, length, quality score, genotype, and other relevant attributes. The quality scores were available from Sniffles, DeBreak, and SVIM, while cuteSV and pbsv did not provide scores. We merged and indexed the SVs according to the procedure described in Section 1 of Supporting Information, resulting in 16,044 positions. We then evaluated three Bayesian methods: Model‐0.950, Model‐0.990, and Model‐0.999. Figure 3 illustrates the distribution of PEPs estimated by the model, along with the corresponding FDR curve at different thresholds. The quality score distributions were fitted and depicted in Section 3 of the Supporting Information.

For comparison with other integration methods, we added the latest tool combiSV (Dierckxsens et al. 2021), which used benchmark datasets to evaluate the performance of different tools and then combined the SVs. Specifically, combiSV conducted an analysis of each SV detection tool, focusing on how false positive rates correlate with the accuracy of various statistics, including position, SV type, and genotype for each SV and genotype. In cases where multiple callers identify the same SV, combiSV will prioritize one caller for each statistic based on the results of the analysis of the simulated benchmark. For example, pbsv will be prioritized for its accuracy in position and length statistics, whereas cuteSV will take precedence for genotype accuracy. In Table 4, we observed that the Bayesian method, specifically Model‐0.990 and Model‐0.999, achieved the highest F1 scores compared to individual tools and other combination methods. However, biases remained in the estimation of FDR, with all the results underestimated. Despite the biases, the relative order of the estimates was preserved. We have investigated possible reasons in Section 3.1, arguing that these biases could be attributed to suboptimal score distributions, as evidenced in Section 3 of the Supporting Information.

**TABLE 4:** Comparison results for the simulated Pacbio‐like sequencing data. The bold number indicates the best F1 score.

Tool/method	Number of SVs	1‐$\hat{{\mathrm{FDR}}_{j}}$	Precision	Recall	F1 score
cuteSV	9,052	0.916	0.859	0.830	0.844
pbsv	7,895	0.945	0.893	0.776	0.831
Sniffles	9,524	0.920	0.817	0.849	0.833
DeBreak	8,952	0.932	0.848	0.838	0.843
SVIM	14,900	0.582	0.542	0.872	0.670
Model‐0.950	9,400	0.950	0.824	0.847	0.835
Model‐0.990	8,784	0.990	0.869	0.838	0.853
Model‐0.999	8,291	0.999	0.902	0.824	**0.861**
combiSV	7,501	0.925	0.924	0.760	0.834
Vote 1	9,046	0.947	0.849	0.846	0.847
Vote 2	6,965	0.995	0.939	0.726	0.819

In fact, combiSV was unable to cover the latest tool DeBreak. To ensure a fair comparison, we conducted an additional analysis comparing combiSV with our model using only the same four tools, excluding DeBreak. The results are presented in Table 5. We observed differences in the estimated precision (1‐${\hat{\text{FDR}}}_{j}$) compared to the values in Table 4, indicating that the accuracy can vary depending on the specific input data. In this comparison, Model‐0.990 outperformed all other methods, although it performed slightly worse than Model‐0.999 from Table 4, as it no longer incorporated information from DeBreak.

**TABLE 5:** Comparison results for the simulated Pacbio‐like sequencing data when integrating four tools: cuteSV, pbsv, Sniffles, and SVIM. The bold number indicates the best F1 score.

Tool/method	number of SVs	1‐$\hat{{\mathrm{FDR}}_{j}}$	Precision	Recall	F1 score
cuteSV	9,052	0.923	0.859	0.830	0.844
pbsv	7,895	0.952	0.893	0.776	0.831
Sniffles	9,524	0.925	0.817	0.849	0.833
SVIM	14,900	0.591	0.542	0.872	0.670
Model‐0.950	9,459	0.950	0.822	0.850	0.836
Model‐0.990	8,876	0.990	0.867	0.843	**0.855**
Model‐0.999	8,392	0.999	0.882	0.816	0.848
combiSV	7,501	0.930	0.924	0.760	0.834
Vote 1	8,611	0.972	0.872	0.828	0.849
Vote 2	7,092	0.999	0.933	0.734	0.822

This example further highlights the flexibility of our model in quantifying the detected SVs, while current tools often focus on precision by employing stricter filtering criteria or recall by relaxing the criteria. Our approach addresses this limitation by utilizing quality scores to estimate the FDR, and, in practice, we also recommend using different thresholds for the Bayesian models to suit specific analysis requirements and strike a balance between precision and recall.

We further conducted a real‐data experiment on the Genome in a Bottle Consortium HG002 dataset, utilizing the v0.6 sequence‐resolved SV calls provided by Zook et al. (2020). The dataset contains a benchmark set of INSs and DELs of sizes 50 bp or larger constructed by a majority‐vote type of integrating method from 19 variation callers. Note that this is not a comprehensive evaluation since the benchmark only contains variations identifiable by most callers, and the ground truth cannot be accurately obtained for real data. We extracted reported SVs of all types from five candidate tools, resulting in a total of 81,908 positions after indexing. Table 6 displays the comparison results, where the precision, recall, and F1 score are obtained using Truvari (English et al. 2022). Similarly, our model demonstrated its capability to capture the underlying confidence levels through its FDR estimates. For the strong variations in the benchmark constructed by a majority‐vote way, it is not surprising that Vote 1 achieved the highest F1 score due to its similar integration logic, while the improvement by our model was not as significant, considering that all methods already exhibited good performance.

**TABLE 6:** Comparison results for the HG002 data. The bold number indicates the best F1 score.

Tool/method	1‐$\hat{{\mathrm{FDR}}_{j}}$	Precision	Recall	F1 score
cuteSV	0.889	0.941	0.862	0.900
pbsv	0.624	0.845	0.842	0.843
Sniffles	0.969	0.941	0.944	0.943
DeBreak	0.975	0.953	0.797	0.844
SVIM	0.660	0.728	0.952	0.825
Model‐0.950	0.950	0.916	0.957	0.936
Model‐0.990	0.990	0.924	0.953	0.938
Model‐0.999	0.999	0.925	0.950	0.938
combiSV	0.870	0.895	0.972	0.932
Vote 1	0.982	0.945	0.943	**0.944**
Vote 2	0.999	0.981	0.668	0.795

## 4 Discussion

In this study, we have developed a Bayesian integration model for detecting SVs by combining SV calls from multiple tools. Our key contributions lie in constructing a confidence measure through PEP estimation and FDR control, thereby informing users about the quality of their results. Compared to other integration methods, the advantage of our model is its flexibility in selecting the desired level of precision or recall by adjusting the FDR threshold.

From a statistical modeling perspective, we have addressed the challenge of defining negatives in the SV context by assuming a joint distribution of the observed index data Y and the count data X. This approach can be generalized to other applications where findings are only recognized upon observation, without information about negative cases. It is essentially applicable for integrating any callers which report the same kind of signals from the same dataset, as long as the individual callers' results can be arranged into a matrix of indicators $Y=\{y_{ij}\}$ and a matrix of scores $S=\{s_{ij}\}$, allowing missing values.

The performance of our approach is reliant on the individual tool's effectiveness and the accuracy of the scores in reflecting the quality of SVs. As long as each individual caller does reasonable work and different callers are not strongly correlated, our model will be helpful. We have demonstrated how deviations from the model's assumptions can impact the accuracy of FDR estimation. Therefore, we recommend that future SV callers provide informative quality scores to benefit the research community. Another potential solution involves incorporating training datasets as prior information in the Bayesian model, which may allow us to adjust the posterior error probabilities and reduce biases of the FDR estimates accordingly. In light of the individual posterior error probabilities, that is, $P(z_{i}=0|\cdot )$, it has provided a way to evaluate and compare each called SV with others. Moreover, throughout our simulation and real data analyses, it is noted that the performance with the FDR level being 0.05 (Model‐0.950) is reasonably satisfactory and can be recommended for practical use. Meanwhile, we again remind that practitioners can select the FDR level desired for their own data and settings. All these areas present avenues for our future research.

Our model considers all types and positions of SVs together, which may require more computational time when handling larger datasets. Users have the option to perform separate analyses based on SV types and chromosomes, which can be particularly beneficial when the scores assigned to SVs do not equally reflect their quality across different types or chromosomes.

There are several directions to improve our method. First, our current method does not deal with phasing. Second, if the number of positions n is too big, our Bayesian method based on MCMC can be slow. A divide‐and‐conquer strategy may be helpful. Last but not least, the integration method, which combines results from existing tools, relies on individual callers' output and therefore does not yield unique findings. Our merging procedure also differs from those inter‐sample merging approaches, such as Jasmine (Kirsche et al. 2023) and PanPop (Zheng et al. 2024), which focus on merging multiallelic SVs into informative biallelic variants within each sample. Nonetheless, there is potential to apply our model to provide confidence levels for these approaches as well.

## Authors Contributions

SL and JS conceived the idea and experiments; SL and JH analyzed the human genomic datasets; SL and ZZ implemented the tool‐aware merging approach; SL conducted the modeling and wrote the manuscript; YL, TH, and DW provided valuable insights; XF, SW, and WY coordinated the project. All authors read and approved the final version of the manuscript.

## Funding

The work was supported by grants 7015‐23G, T12‐101/23‐N, R4012‐18, and MHP/033/20 from the HKSAR government and the National Key Research and Development Program of China (Grant No. 2021YFE0203200).

## Conflicts of Interest

The authors declare no conflicts of interest.

## Open Research Badges

This article has earned an Open Data badge for making publicly available the digitally‐shareable data necessary to reproduce the reported results. The data is available in the Supporting Information section.

This article has earned an open data badge “**Reproducible Research**” for making publicly available the code necessary to reproduce the reported results. The results reported in this article could fully be reproduced.

## Supporting information

Supporting Information

Supporting Information

## Data Availability

All related codes and an illustrative example for the simulation data are available on GitHub at https://github.com/ShhawnLian/BayesInteGration.
