# Supplementary material for: Bayesian Integrative Detection of Structural Variations With False Discovery Rate Control
Source: Biom J. 2026 Mar 27;68(2):e70128. doi: 10.1002/bimj.70128 (PMC13022811; doi:10.1002/bimj.70128)
Supplement: Supplementary file 1 — Supporting Information [file BIMJ-68-e70128-s002.pdf]

Supplementary materials for ”Bayesian integrative detection of structural variations with false discovery rate control”

Sheng Lian*[1], Jiandong Shi†[1], Jingyu Hao[1], Zhen Zhang[1], Yongyi Luo[2], Taobo Hu[3], Depeng Wang[4], Xiaodan Fan‡[2], Shu Wang§[3], and Weichuan Yu¶[1]

[1]Department of Electronic and Computer Engineering, The Hong Kong University of Science and Technology, Clear Water Bay, Hong Kong, China.

[2]Department of Statistics and Data Science, The Chinese University of Hong Kong, Shatin, N.T., Hong Kong, China.

[3]Department of Breast Surgery, Peking University People's Hospital, Beijing, China.

[4]GrandOmics Inc., Beijing, China.

*Co-first author

†Co-first author

‡Corresponding author: Xiaodan Fan (E-mail: xfan@cuhk.edu.hk)

§Corresponding author: Shu Wang (E-mail: shuwang@pkuph.edu.cn)

¶Corresponding author: Weichuan Yu (E-mail: eeyu@ust.hk)

1 Tool-aware SV merging

To account for the slight deviations in the positions and lengths for the same structural variations (SVs) detected by different tools, we propose a tool-aware SV merging procedure. The goal is to combine inter-tool SVs while preserving all intra-tool SVs.

The common criteria for comparing two SVs are as follows: two SVs are considered identical if they are located on the same chromosome, share the same type, their starting positions fall within a range of L base pairs, and their length ratio falls between $\frac{1}{R}$ and R. Here, $L = 1,000$ and $R = 2.0$ as used in [1]. However, the process is more complicated when dealing with multiple tools. If one considers conducting pairwise comparisons using the above criteria, the order becomes crucial.

To address this issue, we adopted an interval merging approach that enables the simultaneous merging of all candidate SVs based on their the starting positions and lengths. Specifically, we constructed two intervals for each SV: the position interval $[start_i, start_i + L]$, and the length interval $[length_i, length_i * R]$. The merging process take places if the corresponding intervals overlap, and we avoid merging intervals from the same tool to retain intra-tool SVs. In addition, we use a narrower setting of $L = 500$ and $R = 1.5$ as we are merging multiple tools.

Finally, the resulting sub-intervals represent distinct SVs, and we record the index information of each tool accordingly.

2 Bayesian inference

We develop an efficient Markov Chain Monte Carlo (MCMC) algorithm to perform Bayesian inference [2, 3]. First, conjugate prior distributions are assigned to the parameters:

$$P(z_i = 1) = \lambda \sim \text{Beta}(\alpha, \beta),$$

$$\boldsymbol{\omega}_1 = (\omega_{11}, \omega_{12}, \ldots, \omega_{1J}) \sim \text{Dirichlet}(\theta_{11}, \theta_{12}, \ldots, \theta_{1J})\mathbb{1}_{\omega_{11}<\omega_{12}<\cdots<\omega_{1J}},$$

$$\boldsymbol{\omega}_0 = (\omega_{01}, \omega_{02}, \ldots, \omega_{0J}) \sim \text{Dirichlet}(\theta_{01}, \theta_{02}, \ldots, \theta_{0J})\mathbb{1}_{\omega_{01}>\omega_{02}>\cdots>\omega_{0J}},$$

where "$\mathbb{1}$" denotes the indicator function that evaluates whether the specific condition is satisfied. By default, we assume non-informative priors by setting $\alpha = \beta = 1$ and $\theta_{1j} = \theta_{0j} = 1$, for $1 \leq j \leq J$. For parameters

associated with quality scores, we focus on $j \in \mathcal{A}$ and give

$$\begin{bmatrix} \mu_{1j} \\ \mu_{0j} \end{bmatrix} \sim N\left(\begin{bmatrix} \xi_{1j} \\ \xi_{0j} \end{bmatrix}, \begin{bmatrix} \epsilon_{1j}^2 & 0 \\ 0 & \epsilon_{0j}^2 \end{bmatrix} \right) \mathbb{1}_{\mu_{1j} > \mu_{0j}},$$

$$\frac{1}{\sigma_{1j}^2} \sim \text{Gamma}(\delta_{1j}, \eta_{1j}), \quad \frac{1}{\sigma_{0j}^2} \sim \text{Gamma}(\delta_{0j}, \eta_{0j}).$$

Our regular settings are $\xi_{1j} = b_j$, $\xi_{0j} = a_j$, and $\epsilon_{1j}^2 = \epsilon_{0j}^2 = 1$. If the scores are scaled to have a unit variance, we can set $\delta_{1j} = \delta_{0j} = 2$, $\eta_{1j} = \eta_{0j} = 1$. All these hyperparameters are flexible, and users can adjust these values according to specific cases.

We collect the parameters as $\boldsymbol{\Theta} = \{\boldsymbol{\omega}_1, \boldsymbol{\omega}_0, \lambda, \boldsymbol{\mu}_1 = \{\mu_{1j}\}_{j\in\mathcal{A}}, \boldsymbol{\mu}_0 = \{\mu_{0j}\}_{j\in\mathcal{A}}, \boldsymbol{\sigma}_1^2 = \{\sigma_{1j}^2\}_{j\in\mathcal{A}}, \boldsymbol{\sigma}_0^2 = \{\sigma_{0j}^2\}_{j\in\mathcal{A}}, \boldsymbol{v} = \{v_{jk}\}_{j=1,\ldots,J;k=1,\ldots,J}\}$. The complete-data likelihood function can be expressed as

$$p(\boldsymbol{Y}, \boldsymbol{X}, \boldsymbol{S}, \boldsymbol{O}, \boldsymbol{Z}|\boldsymbol{\Theta}) = p(\boldsymbol{S}, \boldsymbol{O}|\boldsymbol{Z}, \boldsymbol{Y}, \boldsymbol{\mu}_1, \boldsymbol{\mu}_0, \boldsymbol{\sigma}_1^2, \boldsymbol{\sigma}_0^2) p(\boldsymbol{Y}|\boldsymbol{X}, \boldsymbol{v}) p(\boldsymbol{X}|\boldsymbol{Z}, \boldsymbol{\omega}_1, \boldsymbol{\omega}_0) p(\boldsymbol{Z}|\lambda).$$

Along with the priors, we derive the full conditional posterior distributions for the unknown parameters and missing data in the following, where an efficient sampling procedure is designed. Without loss of generality, we denote "$|\cdot$" as conditioning on all other quantities.

Let $q_i = P(z_i = 1|\cdot)$. For $\{z_i\}_{i=1,\ldots,n}$, and λ, we have

$$\lambda|\cdot \sim \text{Beta}(\sum_{i=1}^n z_i + \alpha, \sum_{i=1}^n (1 - z_i) + \beta),$$

$$z_i|\cdot \sim \text{Binomial}(q_i = \frac{p_{1i}}{p_{1i} + p_{0i}}),$$

where $p_{1i}|\cdot = \lambda\omega_{1,x_i} \prod_{j\in\mathcal{A}} \Phi(\frac{a_j - \mu_{1j}}{\sigma_{1j}})^{\mathbb{1}_{s_{ij} \le a_j}} \phi(\frac{s_{ij} - \mu_{1j}}{\sigma_{1j}})^{\mathbb{1}_{a_j < s_{ij} < b_j}} (1 - \Phi(\frac{a_j - \mu_{1j}}{\sigma_{1j}}))^{\mathbb{1}_{s_{ij} \ge b_j}}$, and $p_{0i}|\cdot = (1 - \lambda)\omega_{0,x_i} \prod_{j\in\mathcal{A}} \Phi(\frac{a_j - \mu_{0j}}{\sigma_{0j}})^{\mathbb{1}_{s_{ij} \le a_j}} \phi(\frac{s_{ij} - \mu_{0j}}{\sigma_{0j}})^{\mathbb{1}_{a_j < s_{ij} < b_j}} (1 - \Phi(\frac{a_j - \mu_{0j}}{\sigma_{0j}}))^{\mathbb{1}_{s_{ij} \ge b_j}}$. Here, $\phi(\cdot)$ and $\Phi(\cdot)$ are density function and cumulative distribution function of standard Gaussian distribution, respectively.

For $\boldsymbol{\omega}_1$ and $\boldsymbol{\omega}_0$, we have

$$p(\boldsymbol{\omega}_1|\cdot) \propto \text{Dirichlet}(\boldsymbol{\theta}_1') \mathbb{1}_{\omega_{11} < \omega_{12} < \cdots < \omega_{1J}},$$

$$p(\boldsymbol{\omega}_0|\cdot) \propto \text{Dirichlet}(\boldsymbol{\theta}_0') \mathbb{1}_{\omega_{01} > \omega_{02} > \cdots > \omega_{0J}},$$

where $\theta_{1k}' = \theta_{1k} + \sum_{i=1}^n \mathbb{1}_{x_i = k, z_i = 1}$, and $\theta_{0k}' = \theta_{0k} + \sum_{i=1}^n \mathbb{1}_{x_i = k, z_i = 0}$, for $k = 1, \ldots, J$. However, it is

not efficient to sample these restricted Dirichlet distributions using a simple Metropolis step. Instead, we employ a procedure that involves sampling restricted Gamma variables first and then transforming them into the target parameters [4, 5]. Specifically, the strategy is to simulate $(\gamma_{11}, \gamma_{12}, \dots, \gamma_{1J})$ with density $\text{Gamma}(\boldsymbol{\theta}_1', 1)\mathbb{1}_{\gamma_{11}<\gamma_{12}<\cdots<\gamma_{1J}}$, and $(\gamma_{01}, \gamma_{02}, \dots, \gamma_{0J})$ with density $\text{Gamma}(\boldsymbol{\theta}_0', 1)\mathbb{1}_{\gamma_{01}>\gamma_{02}>\cdots>\gamma_{0J}}$, and then set $\omega_{1k}|\cdot = \frac{\gamma_{1k}}{\sum_{k=1}^J \gamma_{1k}}$ and $\omega_{0k}|\cdot = \frac{\gamma_{0k}}{\sum_{k=1}^J \gamma_{0k}}$ for $k = 1, \dots, J$. The gamma variables are drawn iteratively using a Gibbs sampling approach from the conditional distributions:

$$\gamma_{1k}|\cdot \sim \text{Gamma}(\theta_{1k}', 1)\mathbb{1}_{\gamma_{1,k-1}<\gamma_{1k}<\gamma_{1,k+1}},$$

$$\gamma_{0k}|\cdot \sim \text{Gamma}(\theta_{0k}', 1)\mathbb{1}_{\gamma_{0,k-1}>\gamma_{0k}>\gamma_{0,k+1}},$$

for $k = 1, \dots, J$, where $\gamma_{10} = -\infty$, $\gamma_{1,J+1} = \infty$, $\gamma_{0,0} = \infty$, and $\gamma_{0,J+1} = -\infty$. These truncated gamma distributions are samples based on the method described in [6].

For the parameters relates to the scores, we focus on $j \in \mathcal{A}$, and the conditional distribution are as follows:

$$o_{ij}|\cdot \begin{cases} \sim N(\mu_{1j}, \sigma_{1j}^2)^{\mathbb{1}_{z_i=1,y_{ij}=1}} N(\mu_{0j}, \sigma_{0j}^2)^{\mathbb{1}_{z_i=0,y_{ij}=1}} \mathbb{1}_{o_{ij}\le a_j}, & \text{if } s_{ij} = a_j \\ = s_{ij}, & \text{if } a_j < s_{ij} < b_j \\ \sim N(\mu_{1j}, \sigma_{1j}^2)^{\mathbb{1}_{z_i=1,y_{ij}=1}} N(\mu_{0j}, \sigma_{0j}^2)^{\mathbb{1}_{z_i=0,y_{ij}=1}} \mathbb{1}_{o_{ij}\ge b_j}, & \text{if } s_{ij} = a_j \end{cases},$$

$$\mu_{1j}|\cdot \sim N\left(\frac{\sum_{i:z_i=1,y_{ij}=1} o_{ij}/\sigma_{1j}^2 + \xi_{1j}/\epsilon_{1j}^2}{\sum_i \mathbb{1}_{z_i=1,y_{ij}=1}/\sigma_{1j}^2 + 1/\epsilon_{1j}^2}, \frac{1}{\sum_i \mathbb{1}_{z_i=1,y_{ij}=1}/\sigma_{1j}^2 + 1/\epsilon_{1j}^2}\right),$$

$$\frac{1}{\sigma_{1j}^2}|\cdot \sim \text{Gamma}(\frac{1}{2}\sum_i \mathbb{1}_{z_i=1,y_{ij}=1} + \delta_{1j}, \frac{1}{2}\sum_{i:z_i=1,y_{ij}=1}(o_{ij} - \mu_{1j})^2 + \eta_{1j}),$$

$$\mu_{0j}|\cdot \sim N\left(\frac{\sum_{i:z_i=0,y_{ij}=1} o_{ij}/\sigma_{0j}^2 + \xi_{0j}/\epsilon_{0j}^2}{\sum_i \mathbb{1}_{z_i=0,y_{ij}=1}/\sigma_{0j}^2 + 1/\epsilon_{0j}^2}, \frac{1}{\sum_i \mathbb{1}_{z_i=0,y_{ij}=1}/\sigma_{0j}^2 + 1/\epsilon_{0j}^2}\right),$$

$$\frac{1}{\sigma_{0j}^2}|\cdot \sim \text{Gamma}(\frac{1}{2}\sum_i \mathbb{1}_{z_i=0,y_{ij}=1} + \delta_{0j}, \frac{1}{2}\sum_{i:z_i=0,y_{ij}=1}(o_{ij} - \mu_{0j})^2 + \eta_{0j}).$$

With regard to the condition $\mu_{1j} > \mu_{0j}$, we perform relabeling that permutes the sample (μ_{1j}, μ_{0j}) in each iteration accordingly to ensure this constraint is satisfied [7].

Lastly, we assign a prior distribution of $\text{Dirichlet}(\theta_1^{(v)}, \theta_2^{(v)}, \cdots, \theta_J^{(v)})$ to $(v_{1k}, v_{2k}, \cdots, v_{Jk})$ for all $k = 1, \dots, J$, with the default values of $\theta_j^{(v)} = 1$ for $j = 1, \dots, J$. Consequently, the corresponding posterior distribution can be derived as $(v_{1k}, v_{2k}, \cdots, v_{Jk}) \sim \text{Dirichlet}(\theta_{1k}'^{(v)}, \theta_{2k}'^{(v)}, \cdots, \theta_{Jk}'^{(v)})$, where $\theta_{jk}'^{(v)} = \sum_{i=1}^n \mathbb{1}_{x_i=k,y_{ij}=1} + \theta_j^{(v)}$, for $j = 1, \dots, J$. However, since this estimation is not the main focus of our study

and it does not impact the estimation of other parameters, we can directly estimate them independently using maximum likelihood estimation by $\hat{v}_{jk} = \frac{\sum_{i=1}^{n} \mathbb{1}_{x_i=k, y_{ij}=1}}{\sum_{i=1}^{n} \mathbb{1}_{x_i=k}}$.

3 Quality score distributions

In this section, we present the score distributions of Sniffles, DeBreak, and SVIM using the simulated PacBio-like sequencing data. Both the fitted uncensored and censored Gaussian distribution were plotted in Figure 1, with boundary sets (a_j, b_j) set as $(-\infty, 60)$, $(-\infty, 60)$, $(0, \infty)$, for Sniffles, DeBreak, and SVIM, respectively. Additionally, we separated the mixture components, where component 1 represented $N(\mu_{1j}, \sigma_{1j}^2)$ and component 0 represented $N(\mu_{0j}, \sigma_{0j}^2)$. The fitting results were satisfactory, clearly distinguishing the two components as assumed by our model.

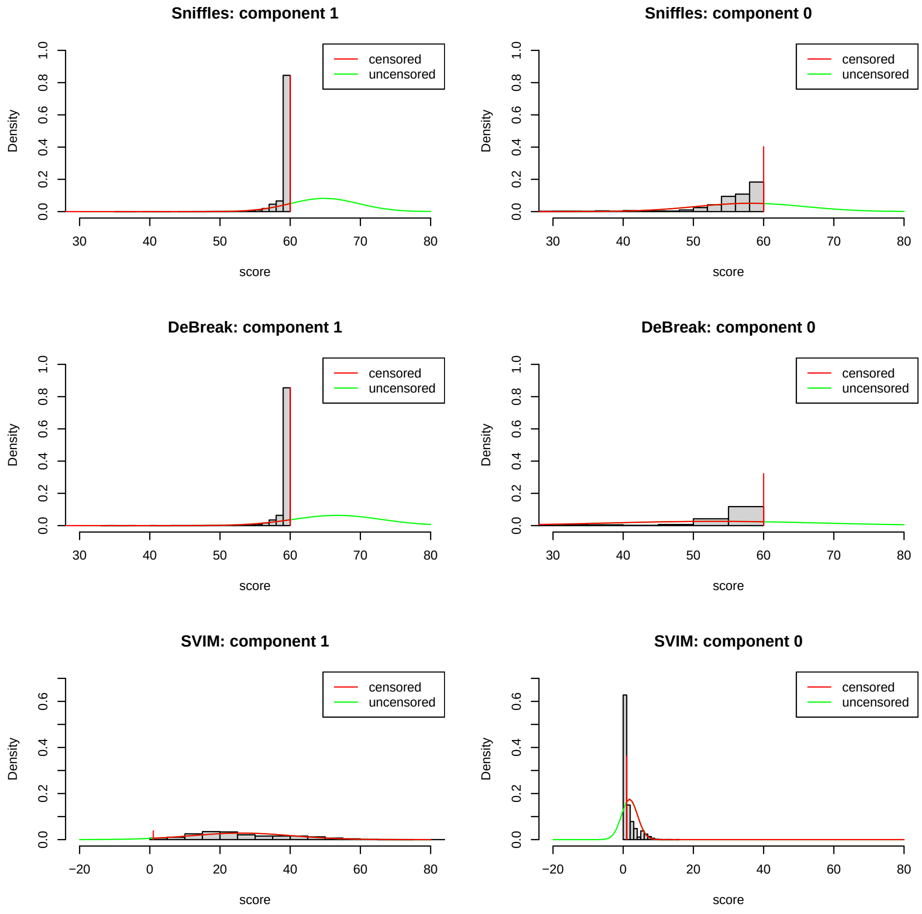

Figure 1: Fitting of the quality score distributions.

Using the ground truth information, we further separated the true positives and false positives and depicted the score distributions of the three methods in Figure 2. It is observed that while the scores

from Sniffles and DeBreak exhibit differences between true positives and false positives, the gap for SVIM is insignificant. This observation is ironic because SVIM is the only method that intentionally designed a quality score, accounting for factors such as supporting read count, span deviation, and position deviation. In contrast, both Sniffles and DeBreak merely report the mean mapping quality of the supporting reads. Such discrepancy from our assumption that a larger score for a reported SV indicates a higher probability of being a true positive has introduced bias into the performance of our model.

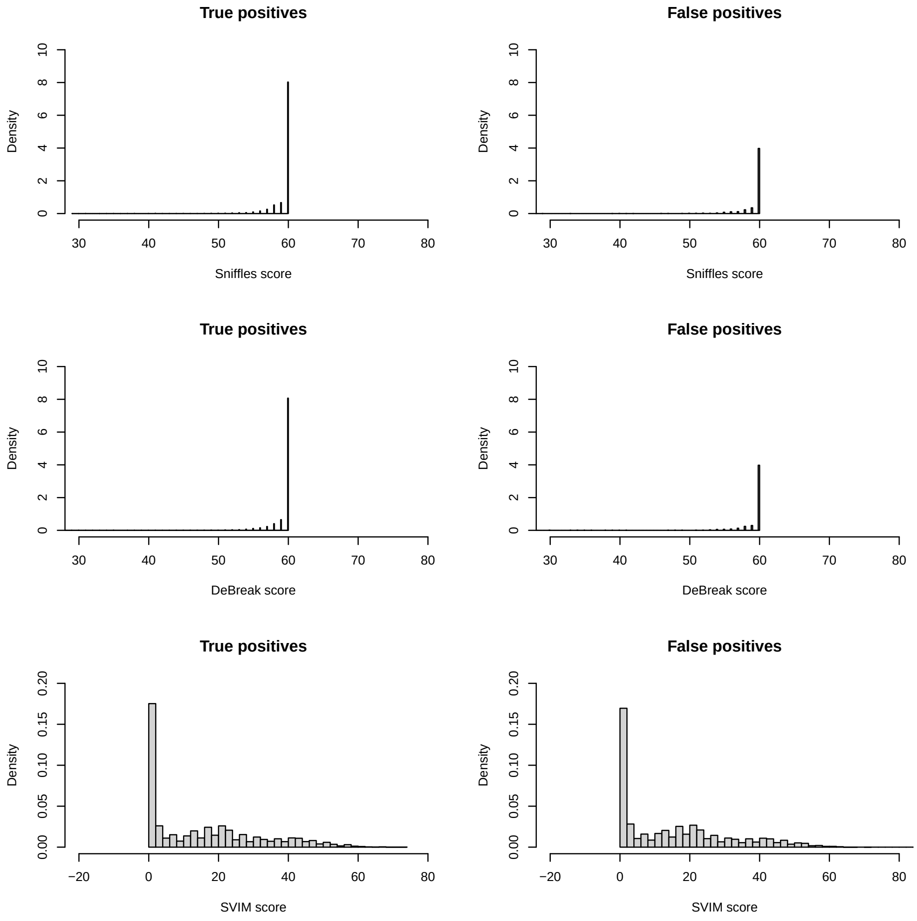

Figure 2: Score distributions with respect to true positives and false positives.

4 Additional analyses with NA19238 and NA19239

We further consider two additional real datasets (NA19238 and NA19239) to illustrate the usefulness of our proposed method [8]. We follow the same procedure as we did for HG002 while an important issue is that there are no benchmark SV set for these two data sets. Thus a comparison of recall and precision is not

available. We only report the numbers of SV callings and their estimated FDR by our proposed method in Table 1. It is as expected that our new method again controls the estimated FDR well and shows the charm of adjusting the FDR level continuously according to practitioners' flexible needs.

Table 1: Comparison results for the NA19238 and NA19239.

Tool/Method	NA19238		NA19239	
	number of SVs	1-$\hat{\text{FDR}}_j$	number of SVs	1-$\hat{\text{FDR}}_j$
cuteSV	23392	0.901	23799	0.902
pbsv	50690	0.434	50659	0.442
Sniffles	20428	0.962	21024	0.958
Debreak	14642	0.953	14979	0.951
SVIM	60545	0.435	60793	0.441
Model-0.950	26189	0.950	26678	0.950
Model-0.990	22944	0.990	23437	0.990
Model-0.999	19380	0.999	19880	0.999
Vote 1	21783	0.960	22264	0.957
Vote 2	12148	0.990	12439	0.991

References

[1] Chen Y, Wang AY, Barkley CA, Zhang Y, Zhao X, Gao M, et al. Deciphering the exact breakpoints of structural variations using long sequencing reads with DeBreak. Nature Communications. 2023;14(1):283.

[2] Gelman A, Carlin JB, Stern HS, Rubin DB. Bayesian Data Analysis. 2nd ed. Chapman and Hall/CRC; 2004.

[3] Robert CP, Casella G. Monte Carlo Statistical Methods (Springer Texts in Statistics). Berlin, Heidelberg: Springer-Verlag; 2005.

[4] Devroye L. Sample-based non-uniform random variate generation. In: Proceedings of the 18th conference on Winter simulation; 1986. p. 260-5.

[5] Sarafoglou A, Haaf JM, Ly A, Gronau QF, Wagenmakers EJ, Marsman M. Evaluating multinomial order restrictions with bridge sampling. Psychological Methods. 2023;28(2):322.

[6] Nadarajah S, Kotz S. R Programs for Truncated Distributions. Journal of Statistical Software, Code Snippets. 2006;16(2):1–8.

[7] Stephens M. Dealing with label switching in mixture models. Journal of the Royal Statistical Society: Series B (Statistical Methodology). 2000;62(4):795-809.

[8] Consortium GP. A map of human genome variation from population scale sequencing. Nature. 2010;467(7319):1061.
