# Supplementary material for: Bayesian Integrative Detection of Structural Variations With False Discovery Rate Control
Source: Biom J. 2026 Mar 27;68(2):e70128. doi: 10.1002/bimj.70128 (PMC13022811; doi:10.1002/bimj.70128)
Supplement: Supplementary file 2 — Supporting Information [file BIMJ-68-e70128-s001.zip › Code_and_Data/README.pdf]

Supplement Data and Code
Title: Bayesian integrative detection of structural variations with false discovery rate control

```
R configuration (`sessionInfo()`):
R version 4.5.1 (2025-06-13)
Platform: x86_64-pc-linux-gnu
Running under: Ubuntu 22.04.4 LTS

Matrix products: default
BLAS:   /usr/lib/x86_64-linux-gnu/blas/libblas.so.3.10.0
LAPACK: /usr/lib/x86_64-linux-gnu/lapack/liblapack.so.3.10.0  LAPACK version
3.10.0

locale:
 [1] LC_CTYPE=en_GB.UTF-8       LC_NUMERIC=C
 [3] LC_TIME=en_HK.UTF-8        LC_COLLATE=en_GB.UTF-8
 [5] LC_MONETARY=en_HK.UTF-8    LC_MESSAGES=en_GB.UTF-8
 [7] LC_PAPER=en_HK.UTF-8       LC_NAME=C
 [9] LC_ADDRESS=C               LC_TELEPHONE=C
[11] LC_MEASUREMENT=en_HK.UTF-8 LC_IDENTIFICATION=C

time zone: Asia/Hong_Kong
tzcode source: system (glibc)

attached base packages:
[1] stats     graphics  grDevices utils     datasets  methods   base

other attached packages:
[1] MCMCpack_1.7-1 MASS_7.3-55    coda_0.19-4.1

loaded via a namespace (and not attached):
 [1] compiler_4.5.1     Matrix_1.7-3       quantreg_6.1       SparseM_1.84-2
 [5] survival_3.2-13    MatrixModels_0.5-4 splines_4.5.1      grid_4.5.1
 [9] mcmc_0.9-8         lattice_0.20-45

Python configuration (`pip list`):
Package          Version
---------------- -----------
bwapy            0.1.4
cffi             1.17.1
edlib            1.3.9.post1
intervaltree     3.1.0
joblib           1.5.2
markdown-it-py   4.0.0
mdurl            0.1.2
networkx         3.4.2
numpy            2.2.6
pandas           2.3.2
pip              25.1
psutil           7.0.0
```

pyabpoa	1.5.4
pycparser	2.22
Pygments	2.19.2
pysam	0.23.3
pytabix	0.1
python-dateutil	2.9.0.post0
pytz	2025.2
pywfa	0.5.1
rich	14.1.0
setuptools	78.1.1
six	1.17.0
sortedcontainers	2.4.0
truvari	5.3.0
tzdata	2025.2
wheel	0.45.1

Code execution process: (computer: Intel(R) Core(TM) i7-9700 CPU @3.00GHz, 3000 Mhz, 8 Cores, 8 Logical Processor(s) with each .R requiring one core)
1.Set folder `Code_and_Data` as the current working directory. All codes set seed as 123.
2.Results of Simulation studies: ~20 minutes
(a)Table 1 (Case 1): run `simulation_case1.R`
(b)Table 2 (Case 2): run `simulation_case2.R`
(c)Table 3 (Case 3): run `simulation_case3.R`
3.Results of the simulated Pacbio-like sequencing data: ~25 minutes
(a)Index procedure: ~1 minute
We have provided the CSV files that included essential SV information from different detection tools in the `./arrayfile_simulated/` folder. We also provided the indexed results in the `./result_simulated_1/` and `./result_simulated_2/` folders, which can be directly used for rerunning our codes without doing the index procedure again. Detail procedure can be found in the next session.
(b)Combine results:
Figure 3 and Table 4: run `simulation_pacbio1.R`
Table 5: run `simulation_pacbio2.R`
4.Results of the real data:
(a)Index procedure: ~1 minute
We have provided the CSV files that included essential SV information from different detection tools in the `./arrayfile_HG002/`, `./arrayfile_NA19238/` and `./arrayfile_NA19239/` folders. We also provided the indexed results in the `./result_HG002/`, `./result_NA19238/` and `./result_NA19239/` folders, which can be directly used for rerunning our codes without doing the index procedure again. Detail procedure can be found in the next session.
(b)Combine results:
HG002: run `Inference_HG002.R` ~2 hours
NA19238 & NA19239: run `Inference_NA19238_NA19239.R` ~4.5 hours
Note that this step present part of the results. For comparison, we use the Truvari tool to obtain precision, recall and F1 score. Detail procedure can be found in the next session.

Source of data:

1.The simulated Pacbio-like sequencing data in the `./Data_simulated/` folder are generated by VarSim and PBSIM2 with parameter setting: Read length (mean 5,000 bp and sd 1,000 bp), accuracy (95%) and sequencing depth (20x).
2.Access GIAB Consorium HG002 Dataset and Human Genome Assembly GRCh37 reference:
```
mkkdir -p ref giab
FTPDIR=ftp://ftp-trace.ncbi.nlm.nih.gov/giab/ftp/data/AshkenazimTrio/analysis/
curl -s ${FTPDIR}/NIST_SVs_Integration_v0.6/HG002_SVs_Tier1_v0.6.bed > giab/HG002_SVs_Tier1_v0.6.bed
curl -s ${FTPDIR}/NIST_SVs_Integration_v0.6/HG002_SVs_Tier1_v0.6.vcf.gz > giab/HG002_SVs_Tier1_v0.6.vcf.gz
curl -s ftp://ftp-trace.ncbi.nih.gov/1000genomes/ftp/technical/reference/phase2_reference_assembly_sequence/hs37d5.fa.gz > ref/human_hs37d5.fasta.gz
gunzip ref/human_hs37d5.fasta.gz
sed -i '/^[^>]/ y/BDEFHIJKLMNOPQRSUVWXYZbdefhijklmnopqrsuvwxyz/NNNNNNNNNNNNNNNNNNNNNNNNNNNNNNNNNNNNNNNNNN/' ref/human_hs37d5.fasta
curl -s https://raw.githubusercontent.com/PacificBiosciences/pbsv/master/annotations/human_hs37d5.trf.bed > ref/human_hs37d5.trf.bed
```
3.Use of Truvari for comparison
(a)Download the GIAB Consorium HG002 Dataset and Human Genome Assembly GRCh37 from Step 2 ;
(b)Convert combine results into VCF files (Take `result_HG002/res_D_0.95.csv` as an example);
```
python vcf.py result_HG002/res_D_0.95.csv result_HG002/res_D_0.95.vcf
bgzip -k result_HG002/res_D_0.95.vcf
tabix result_HG002/res_D_0.95.vcf.gz
```
(c)Truvari
```
truvari bench -b ./giab/HG002_SVs_Tier1_v0.6.vcf.gz -c result_HG002/res_D_0.95.vcf.gz -o result_HG002/res_D_0.95 --dup-to-ins --passonly --includebed ./giab/HG002_SVs_Tier1_v0.6.bed -p 0 -r 1000 -P 0.5
```
4.Index procedure: To get SV infomation (CSV files) from each SV detection tool (VCF files) and to index these SVs.
The index results are provided in the data file for simulation results and do not need to be re-done.
(a)When combining five tools for the Pacbio-like sequencing data:
```
python vcf2arr_simulated/vcf2arr_svim.py
python vcf2arr_simulated/vcf2arr_pbsv.py
python vcf2arr_simulated/vcf2arr_sniffles.py
python vcf2arr_simulated/vcf2arr_debreak.py
python vcf2arr_simulated/vcf2arr_cutesv.py
python vcf2arr_simulated/merge_csv.py`
python vcf2arr_simulated/vcf2arr_gt.py`
```

python vcf2arr_simulated/vcf2arr_combisv.py`
mv arrayfile_simulated/merged.csv arrayfile_simulated/merged_1.csv
mkdir result_simulated_1
python Index_Score.py --csv_path ./arrayfile_simulated/merged_1.csv --output_path ./result_simulated_1/
```
(b)When combining four tools for the Pacbio-like sequencing data:
```
python vcf2arr_simulated/vcf2arr_svim.py
python vcf2arr_simulated/vcf2arr_pbsv.py
python vcf2arr_simulated/vcf2arr_sniffles.py
python vcf2arr_simulated/vcf2arr_cutesv.py
python vcf2arr_simulated/merge_csv.py`
python vcf2arr_simulated/vcf2arr_gt.py`
python vcf2arr_simulated/vcf2arr_combisv.py`
mv arrayfile_simulated/merged.csv arrayfile_simulated/merged_2.csv
mkdir result_simulated_2
python Index_Score.py --csv_path ./arrayfile_simulated/merged_2.csv --output_path ./result_simulated_2/
```
(c)When combining five tools for the HG002 data (similar for the NA19238 and NA19239):
```
python vcf2arr_HG002/vcf2arr_svim.py
python vcf2arr_HG002/vcf2arr_pbsv.py
python vcf2arr_HG002/vcf2arr_sniffles.py
python vcf2arr_HG002/vcf2arr_cutesv.py
python vcf2arr_HG002/merge_csv.py`
python vcf2arr_HG002/vcf2arr_gt.py`
python vcf2arr_HG002/vcf2arr_combisv.py`
mkdir result_HG002
python Index_Score.py --csv_path ./arrayfile_HG002/merged.csv --output_path ./result_HG002/
```

Source of tools:
- VarSim: https://github.com/bioinform/varsim
- PBSIM2: https://github.com/yukiteruono/pbsim2
- SVIM: https://github.com/eldariont/svim
- Sniffles: https://github.com/fritzsedlazeck/Sniffles
- DeBreak: https://github.com/Maggi-Chen/DeBreak
- pbsv: https://github.com/PacificBiosciences/pbsv
- cuteSV: https://github.com/tjiangHIT/cuteSV
- combiSV: https://github.com/ndierckx/combiSV
```
